# Dental pulp exposure, periapical inflammation and suppurative osteomyelitis of the jaws in juvenile Baltic grey seals (*Halichoerus grypus grypus*) from the late 19th century

**DOI:** 10.1371/journal.pone.0215401

**Published:** 2019-04-12

**Authors:** Uwe Kierdorf, Morten T. Olsen, Patricia Kahle, Catharina Ludolphy, Horst Kierdorf

**Affiliations:** 1 Department of Biology, University of Hildesheim, Hildesheim, Germany; 2 Natural History Museum of Denmark, Department of Biology, University of Copenhagen, Copenhagen, Denmark; Ecole Normale Supérieure de Lyon, FRANCE

## Abstract

The systematic analysis of museum collections can provide important insights into the dental and skeletal pathology of wild mammals. Here we present a previously unreported type of dental defect and related skull pathology in five juvenile Baltic grey seals that had been collected in the course of a seal culling program along the Danish coast in 1889 and 1890. All five skulls exhibited openings into the pulp cavities at the crown tips of all (four animals) or two (one animal) canines as well as several incisors and (in one animal) also some anterior premolars. The affected teeth showed wide pulp cavities and thin dentin. Pulp exposure had caused infection, inflammation, and finally necrosis of the pulp. As was evidenced by the extensive radiolucency around the roots of the affected teeth, the inflammation had extended from the pulp into the periapical space, leading to apical periodontitis with extensive bone resorption. Further spreading of the inflammation into the surrounding bone regions had then caused suppurative osteomyelitis of the jaws. The postcanine teeth of the pathological individuals typically had dentin of normal thickness and, except for one specimen, did not exhibit pulp exposure. The condition may have been caused by a late onset of secondary and tertiary dentin formation that led to pulp exposure in anterior teeth exposed to intense wear. Future investigations could address a possible genetic causation of the condition in the studied grey seals.

## Introduction

The systematic analysis of mammalian skeletons from museum collections can provide important information on the spectrum and prevalence of anomalies and diseases of bones and teeth in the studied species [1–4]. The findings of such investigations are not only of interest with respect to comparative pathology, but also helpful in the assessment of population health and its changes over time. The results can thereby contribute to the identification of emerging health threats and to an effective population management.

Several studies reported dental anomalies and lesions, alveolar bone pathology, and degenerative changes of the temporomandibular joint in pinnipeds from a comparative point of view [5–14]. Other studies addressed the prevalence and severity of skull lesions in marine mammals, including polar bears (*Ursus maritimus*) [3], harbor seals (*Phoca vitulina*) [2, 15, 16], and grey seals (*Halichoerus grypus*) [17, 18], in relation to levels of exposure to persistent organic pollutants.

The grey seal is a member of the family Phocidae with a cold temperate to subarctic distribution in North Atlantic waters over the continental shelf [19]. As is typical for the amphibious lifestyle of pinnipeds, it spends parts of its life on land for breeding, molting and thermoregulation. Total population size of the grey seal has more recently been estimated at 632,000 individuals with an increasing trend [19]. The species exhibits a pronounced sexual dimorphism, with adult males from the Eastern North Atlantic weighing between 170 and 310 kg, while the body mass of adult females ranges between 100 and 190 kg [20]. The skull of adult males differs from that of females by its elevated fronto-nasal region [21]. Females reach sexual maturity at 3 to 5 years of age, males at 6 years [21]. Maximum life expectancy in the wild has been estimated at around 35 years for females and 25 years for males [22]. The grey seal is a generalist predator that mainly feeds on a variety of fish species [19, 23]. It uses pierce feeding, i.e., the prey is captured with a piercing bite and then swallowed whole [24].

There exist three geographically separated populations of grey seals, present, respectively, in the western North Atlantic, the Eastern North Atlantic and the Baltic Sea [19, 21]. Currently, two subspecies of the grey seal are recognized, *viz*. the Atlantic grey seal (*Halichoerus grypus atlantica*) and the Baltic grey seal (*H*. *g*. *grypus*) [25, 26].

The population of Baltic grey seals in the early 1900s was estimated at 88,000 to 100,000 individuals, but intensive hunting in the course of a multi-national culling program led to a decline to about 20,000 animals in the 1940s [27, 28]. After cessation of hunting, the population did not recover as expected, but further declined to less than 4,000 individuals in the late 1970s [28]. This further decline was linked to impaired reproductive performance of females caused by exposure to high levels of organochlorines [27, 28]. Declining concentrations of these pollutants in their prey species during recent decades led to an improvement of the health situation of the Baltic grey seals and to an increase in reproductive success and population size, which in 2014 was estimated at between 40,000 and 53,000 individuals [28]. Pupping dates differ among the populations in the Northwest Atlantic, the Northeast Atlantic and the Baltic Sea [19]. Peak pupping in the latter occurs in late February to early March [28]. The period of milk-feeding lasts about 15 to 18 days, and weaning is abrupt. First molting occurs around the time of weaning [28].

As a reaction to an intensifying seal-fishery conflict, a seal culling program was started in Denmark in October 1889 that lasted until 1927 [29, 30]. A bounty system was introduced, and in the years 1889 and 1890 hunters were required to send the skulls of culled seals to the Zoological Museum in Copenhagen (now the Natural History Museum of Denmark) to collect their bounty. Following the introduction of the bounty system, the numbers of grey seals in Danish waters rapidly declined and all breeding colonies disappeared before 1900 [30, 31]. Analysis of mitochondrial DNA indicated that historically the grey seals inhabiting inner Danish waters were part of the large Baltic Sea population [32]. The grey seals currently recolonizing the Kattegat primarily originate from the North Sea population, whereas those recolonizing the southwestern Baltic Sea primarily originate from the population in the central Baltic Sea [32].

Thus far, only few studies have addressed the occurrence of dental anomalies in grey seals [11, 33, 34]. In addition, a recent paper described the dentition of a hybrid between a grey seal and a ringed seal (*Pusa hispida*) [35].

**Fig 1:**
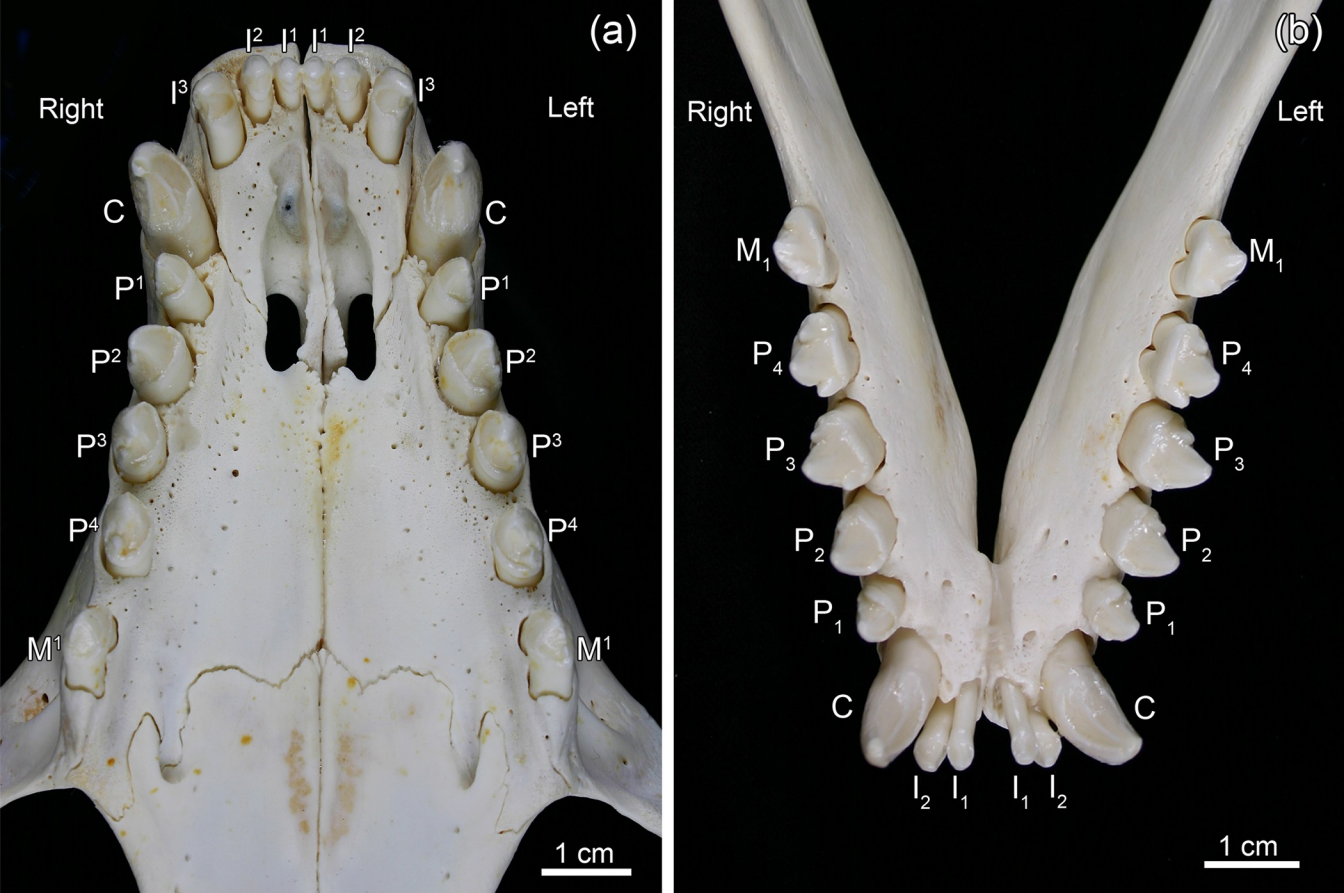
Permanent dentition of a Baltic grey seal (*Halichoerus grypus grypus*; specimen ZMUC CN 1157, juvenile male). (a) Maxillary dentition. (b) Mandibular dentition.

The permanent dentition of the grey seal comprises 34–36 teeth, the dental formula being I 3/2, C 1/1, P 4/4, M 1−2/1 [I: incisors (dentes incisivi), C: canines (dentes canini), P: premolars (dentes praemolares), M: molars (dentes molares] (Fig 1). In the literature on pinnipeds, premolars and molars are often collectively referred to as postcanine teeth [21, 36, 37]. The number of molars can differ between maxilla and mandible, as a maxillary second molar is rather frequently present either uni- or bilaterally. The crowns of the brachydont premolars and molars are of a uniform shape with a large conical central cusp and small secondary cusps (Fig 1). The deciduous teeth of the grey seal are already shed prenatally. Development of all permanent teeth is well advanced at birth, and they erupt a few days after birth [22]. At the end of the short suckling period, the permanent dentition is thus fully erupted and functional.

The bulk of a typical mammalian tooth is formed by dentin that in the crown is covered by enamel and in the root by cementum [37, 38]. While enamel is only formed prior to tooth eruption, deposition of dentin and cementum continues in an incremental fashion during the life of a tooth. In seals, the growth layers in dentin and cementum are used for age estimation [19, 22]. Dentin is formed by odontoblasts that are arranged in a layer at the interface with the dental pulp [38]. The dentin is permeated by dentinal tubules that extend through the entire thickness of the tissue, each dentinal tubule harboring a long odontoblast process [37, 38].

The dentin formed prior to (full) tooth eruption and completion of the apical tooth region (in brachydont teeth) is referred to as primary dentin. It includes the initially formed mantle dentin and the outer zone of the subsequently deposited circumpulpal dentin [39, 40]. Secondary dentin is the regular circumpulpal dentin deposited at a slower rate during the later life of a tooth. [39, 40]. The tubules in secondary dentin are largely continuous with those of the primary dentin, suggesting that both dentin types are formed by the same odontoblasts. Frequently, the direction of the dentinal tubules changes from primary to secondary dentin, and the tubular pattern of secondary dentin is somewhat less regular than that of primary dentin [41]. Tertiary dentin is a more or less irregular tissue formed locally as a pulpal reaction to strong stimuli, such as excessive wear, mechanical, chemical or thermal stresses [39–41]. It has fewer tubules than primary or secondary dentin or may be largely atubular. Tertiary dentin has been classified into reactionary dentin, whose matrix is secreted by remaining postmitotic odontoblasts, and reparative dentin, whose matrix is produced by newly differentiated pulpal cells in response to stronger stimuli following the death of the original odontoblasts [40, 41].

During dentinogenesis first an unmineralized organic matrix, the predentin, is secreted that is subsequently mineralized. The mineral initially forms globular structures referred to as calcospherites that gradually enlarge and thereby become confluent [37, 38, 41]. During later phases of dentinogenesis, when dentin deposition slows down, a more linear pattern of mineralization can often be seen [38]. When the calcospherites fail to fuse, the dentin exhibits unmineralized or hypomineralized areas referred to as interglobular dentin [37, 38].

In the course of a research project on the health of marine mammals from the North Sea and the Baltic Sea, the skulls of grey seals held in the skull collection of the Natural History Museum of Denmark were systematically inspected for dental anomalies and lesions. Here we describe a previously unreported type of dental pathology and its sequelae in the skulls of grey seals collected in the southwestern Baltic region in the period 1889−1890.

## Materials and methods

Three complete and two fragmentary skulls of Baltic grey seals from the skull collection of the Natural History Museum of Denmark, University of Copenhagen, exhibited a similar spectrum of pathological dental and osseous changes. None of the other 204 grey seal skulls from the collection that were inspected in the course of our study showed comparable lesions.

**Fig 2:**
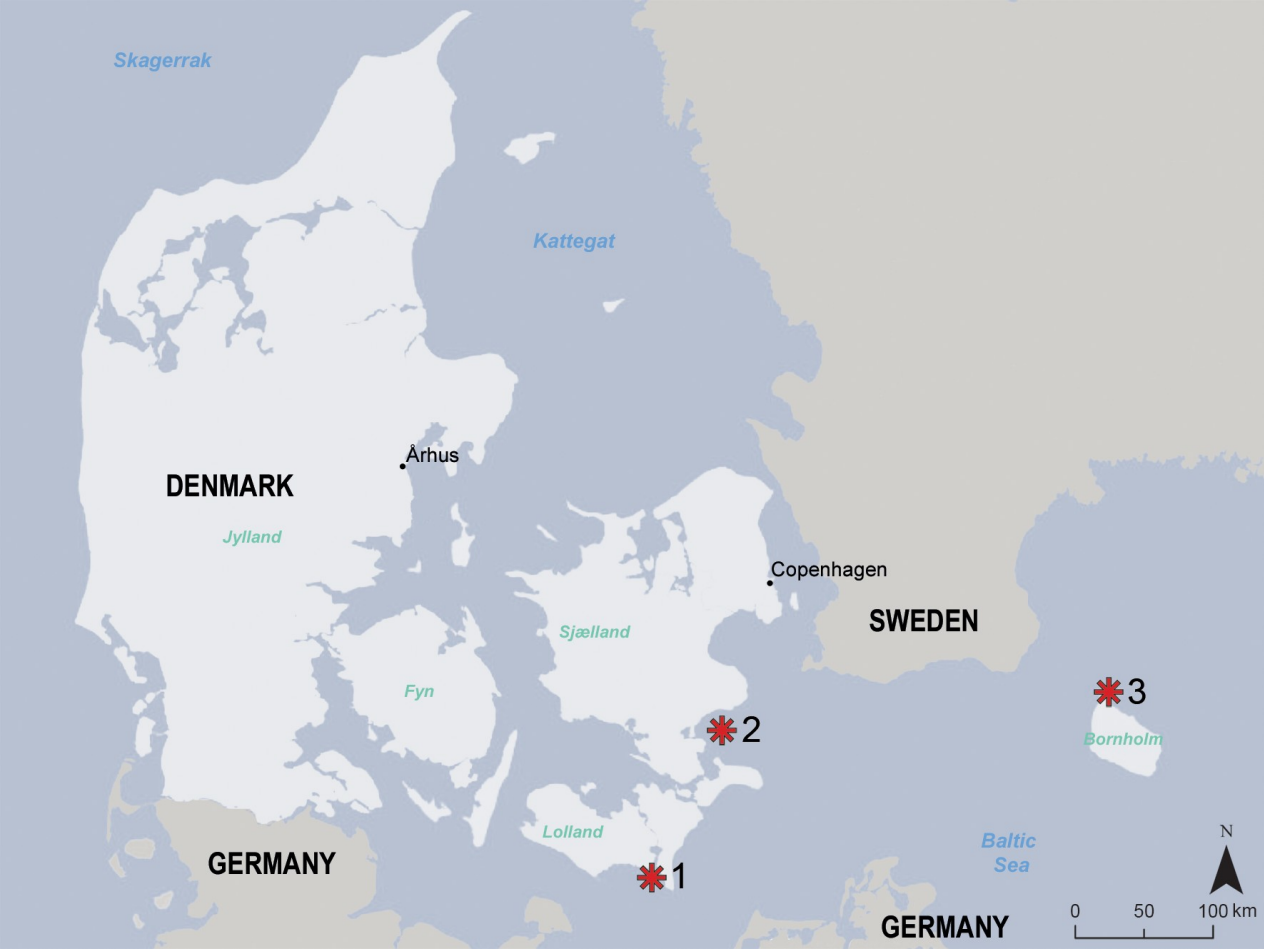
Map indicating the localities (asterisks) where the Baltic grey seals (*Halichoerus grypus grypus*) had been collected in 1889–1890. Five skulls belonged to animals culled on Rødsand sandbank (locality 1), one to an animal culled near Faxe on Sjaelland (locality 2), and one to an animal from Christiansø on Bornholm (locality 3).

The five pathological skulls belonged to individuals that had been collected in the Southwestern Baltic in either 1889 (months October to December) or 1890 (Table 1, Fig 2). In four of the specimens, this information was given both on the skulls and the associated tag. In the fifth specimen (ZMUC 303), this was not the case. However, the specimen’s low ID number, the fact that the skull showed the same type of ink labeling as the other four specimens, and the observation that the handwriting on the piece of paper found in the plastic bag containing the skull was the same as in the case of one of the other four skulls (ZMUC 139, collected in 1890) is taken as evidence that specimen ZMUC 303 was also collected in the said period (probably in 1890). All five skulls are therefore considered to have originated from individuals that had been obtained during the first two years of the aforementioned Danish seal culling program. It is not stated in the records whether the animals had been hunted or were found dead. None of the complete skulls showed evidence of projectile or blunt force (clubbing) trauma. However, two pathological skulls were fragmentary with the entire braincase (ZMUC 139) or large portions of it (ZMUC 303) missing, which would be consistent with a trauma to the skull. Given the origin of the 19th century skulls it is assumed that all originate from grey seals that had been killed in the course of the culling program.

**Table 1:** List of Baltic grey seals (*Halichoerus grypus grypus*) skulls from the collection of the Natural History Museum of Denmark, University of Copenhagen (ZMUC) analyzed in the present study. All teeth present in the specimens belonged to the permanent dentition, and no persistent deciduous teeth were recorded. The labeling is given as it appears on the skulls and the tags (Nobr = November, Dcbr = December).

Identification number, completeness of skull	Labeling on skull / labeling on tag	Sex / age class / condylobasal length	Completeness of dentition (maxillary/mandibular)	Teeth with opened pulp cavities	Signs of inflammatory processes in the jaws
ZMUC 15^5^, complete skull	*Hal*. *gryp*. Rødsand “15^5^”/ *Halichoerus grypus* Rødsand 16-25/10 1889 15.^5^	? / juvenile (young-of-the-year) / 202 mm	maxillary dentition: complete, bilateral presence of a second molar/ mandibular dentition: incomplete; left C, P_1_ and P_2_, and right P_1_ missing due to intravital loss	both I^3^, right mandibular C	yes
ZMUC 42^4^, complete skull	*Hal*. *gryp*. Rødsand 42^4^/ *Halichoerus grypus* Rødsand, Nobr 1889 42.^4^	? / juvenile (young-of-the-year) / 196 mm	maxillary dentition: complete, bilateral presence of an M^2^/ mandibular dentition: complete	all six maxillary I, both I_1_ and all four C	yes
ZMUC 69^3^, complete skull	*Hal*. *gryp*. Rødsand “69^3^”/ *H*. *gryp*. Rødsand, Dcbr. 1889 69.^3^	? / juvenile (young-of-the-year) / 190 mm	maxillary dentition: complete/ mandibular dentition: right I_1_ and both C missing due to intravital loss	all six maxillary I and both maxillary C	yes
ZMUC 139, fragmentary; only rostral part of cranium and both mandibles present	*Hal*. *gryp*. “139” Christiansø/ “139 N VI *Halichoerus grypus* 19/6 90	? / juvenile / nd	maxillary dentition: Both I^1^, both I^2^, left P^1^, and right P^3^ missing due to postmortem loss/ mandibular dentition: left I_1_, I_2_, C and P_1_ missing due to intravital loss; anterior portion of right mandible (including I_1_, I_2_ and C) missing (intravital loss), right P_2_ missing (probably postmortem loss	right I^3^, right maxillary C, right P^1^ and P^2^; left I^3^, left maxillary C and left P^2^	yes
ZMUC 303, Fragmentary; only rostral part of cranium and both mandibles present	*Hal*. *gry*. Faxe “303”/ 303 *Halichoerus grypus* Faxe	? / juvenile / nd	maxillary dentition: complete; mandibular dentition: right P_1_ missing (intravital loss)	all six maxillary I and both maxillary C; right I_1_ and I_2_ and both mandibular C	yes
ZMUC 71^3^, complete skull	*Hal*. *gryp*. Rødsand “71^3^”/ *H*. *gryp*. Rødsand Nobr. 1889 71.^3^	? / juvenile (young-of-the-year) 197 mm	maxillary dentition: complete; mandibular dentition: both P_1_ missing (agenesis)	none	no
ZMUC 71^9^, complete skull	71^9^ H. gryp. Rødsand Dcbr.89./ no tag	?/ juvenile (young-of-the-year) / 199 mm	dentition complete	none	no
ZMUC CN 1157, complete skull	*Halichoerus grypus* ♂ CN 1157/ no tag	male / juvenile / 214 mm	dentition complete	none	no

For comparison, three complete skulls of Baltic grey seals that were free of the lesions seen in the five pathological skulls were also studied (Table 1). Two of the three control skulls (ZMUC 71^3^ and ZMUC 71^9^) had also been collected in 1889 and originated from the same locality (Rødsand, for location see Fig 2) as three of the specimens (ZMUC 15^5^, ZMUC 42^4^, ZMUC 69^3^) exhibiting pathological changes. The third control skull (ZMUC CN 1157) was that of a modern juvenile male grey seal (S1 Fig). No information on the sex of the other seven grey seals was available. As sexual dimorphism in skull size or canine length is marked only in older individuals [22], sex of the juvenile grey seals from the 19th century could not be established. Condylobasal length was measured in the six complete skulls (not possible in the fragmentary skulls ZMUC 139 and ZMUC 303). Values ranged between 190 mm and 202 mm in the five complete skulls from 1889, while condylobasal length in specimen CN 1157 was 214 mm (Table 1).

Based on an assessment of the degree of fusion of sutures and synchondroses, applying the scoring system by Sivertsen [42], condylobasal length (in the complete skulls) and the size of the canines [22], all eight studied skulls were classified as those of juvenile individuals. Three of the pathological skulls (ZMUC 15^5^, ZMUC 42^4^, and ZMUC 69^3^) and two of the control skulls (ZMUC 71^3^ and ZMUC 71^9^) belonged to animals collected in the months October to December (see Table 1 for collection dates). These skulls were classified as those of young-of-the-year. The other three skulls (two pathological, one control) belonged to older juveniles. Specimen ZMUC 139 originated from an individual collected in June 1890 and was characterized by pronounced wear of incisors, canines and anterior premolars. In specimens ZMUC 303 and ZMUC CN 1157, a higher age of the individuals was indicated by the larger size of the (fragmentary or complete) skulls compared to those of the five young-of-the-year. Moreover, in ZMUC CN 1157 the sphenooccipital synchondrosis was already fused over more than 50% of its length, while it was open in all young-of-the-year. Due to the fragmentary condition of the skulls, this synchondrosis could not be assessed in specimens ZMUC 139 and ZMUC 303.

All teeth present in the studied skulls belonged to the permanent dentition, and no persistent deciduous teeth were found. Information on the teeth available for inspection in the specimens is given in Table 1.

The eight skulls were first studied macroscopically. Photos were obtained with a digital camera, and radiographs were taken of all specimens. Subsequently, the right maxillary C (length 33.9 mm) and the right M_1_ of specimen ZMUC 69^3^, the left maxillary C (length 41.2 mm) of specimen ZMUC CN 1157, and the right M_1_ of specimen ZMUC 71^9^ were extracted, photographed, and embedded in epoxy resin (Biodur, Biodur Products, Heidelberg, Germany or Epofix, Struers, Ballerup, Denmark). Canine length of ZMUC 69^3^ falls within the range (32.3 to 38.5 mm) given for 6 to 8 month-old grey seals [22]. The embedded teeth were sectioned longitudinally in the labio-palatal (canines) or the bucco-lingual (molars) plane. The cut surfaces of the resulting halves were smoothed and polished using a series of silicon carbide papers (grits 320−4,000), followed by further sequential polishing on a motorized polisher (Labopol 5, Struers, Ballerup, Denmark) with diamond suspensions of 3 and 1 μm particle size, respectively. Backscattered electron (BSE) imaging of the (uncoated) polished cut surfaces was performed in a scanning electron microscope (SEM, Zeiss Evo Ma 15, Carl Zeiss AG, Oberkochen, Germany), operated in a low pressure mode at 20 kV. Following BSE imaging and hardness testing in the canines (see below), the cut surfaces of the teeth were etched (34% phosphoric acid for 5 seconds) and subsequently repeatedly rinsed with deionized water and propanol. The dried blocks were sputter-coated with gold-palladium (Leica EM ACE200, Leica Microsystems, Wetzlar, Germany), and viewed again in the SEM (at 10 kV) using a secondary electron (SE) detector.

To analyze whether the abnormal condition of the teeth in the five pathological skulls was related to a hypomineralization of their enamel and/or coronal dentin, following BSE imaging and prior to etching we performed hardness testing on the polished cut surfaces of the two extracted canines (from control specimen ZMUC CN 1157 and pathological specimen ZMUC 69^3^). Testing was performed in labial enamel and coronal dentin with a Vickers indenter (load of 25 p, dwell time 20 sec.) in a Dura-Scan 20 hardness tester (Struers, Ballerup, Denmark). The probed surfaces were oriented perpendicular to the tip of the diamond pyramid. In each of the two teeth, eight measurements were performed in enamel (four in the inner half and four in the outer half of the enamel layer) and another eight in dentin (four at a distance of 50 μm and four at a distance of 200 μm from the enamel-dentin junction (EDJ). To improve the visibility of the indentations, prior to testing the probed surfaces were sputter-coated with a very thin (15 nm) layer of gold-palladium. Data for enamel and dentin hardness were compared between the two teeth using the Mann-Whitney U-test, with the level of significance set at p < 0.05.

## Results

**Fig 3:**
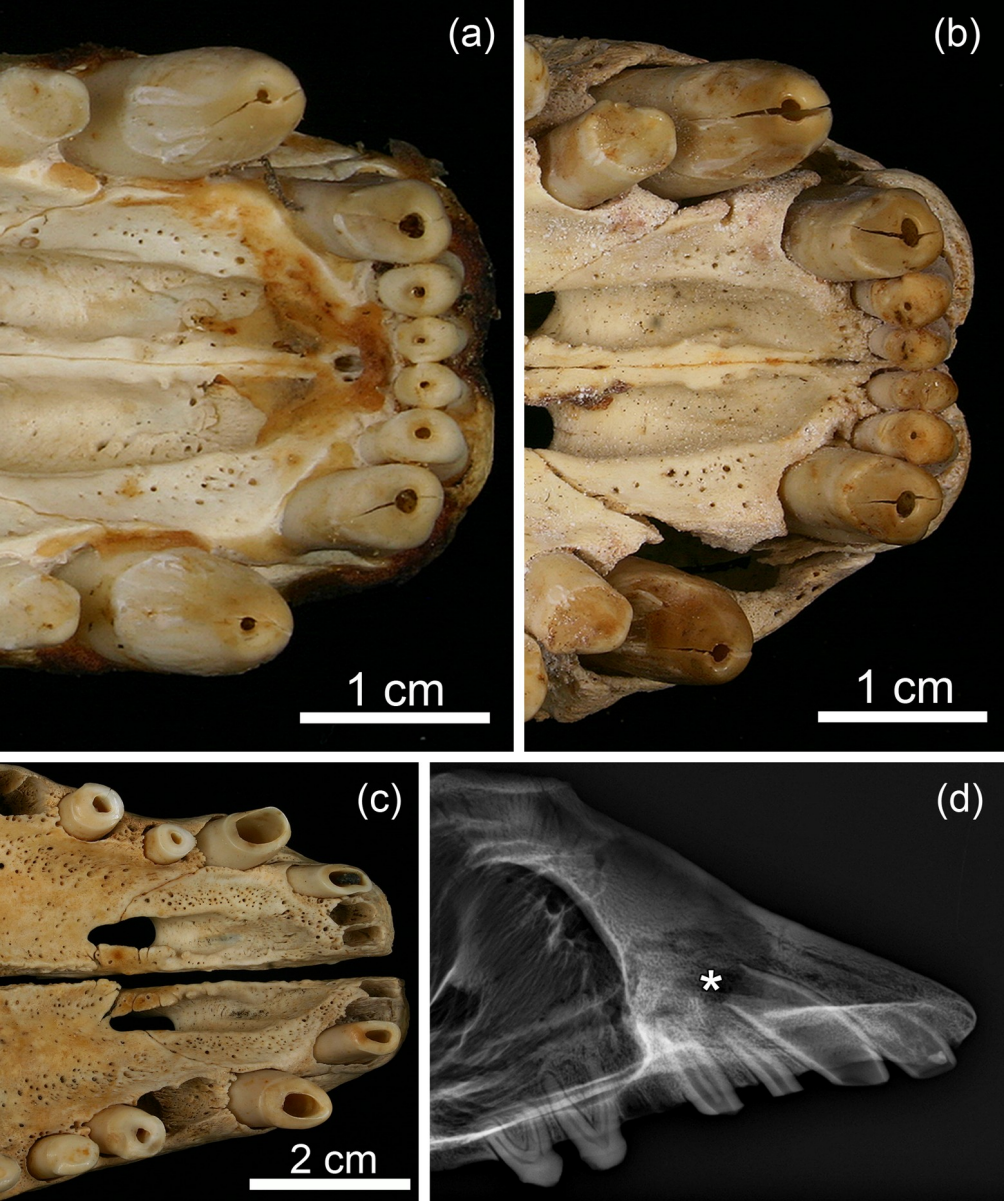
Exposure of the pulp cavities through openings at the crown tips in permanent maxillary teeth of juvenile Baltic grey seals (*Halichoerus grypus grypus*) from the 19th century. **(a)** Ventral view of the incisive and anterior maxillary region of specimen ZMUC 42^4^. Note openings at the crown tips of all six maxillary I and both maxillary C. **(b)** Ventral view of the incisive and anterior maxillary region of specimen ZMUC 69^3^. Note openings at the crown tips of all six I (very small in both I^1^) and both maxillary C. **(c)** Ventral view of the incisive and anterior maxillary region of specimen ZMUC 139. Note openings at the crown tips of both I^3^ and maxillary C, the right P^1^ and P^2^, and the left P^2^ and increased wear of these teeth. Both I^1^, both I^2^, the left P^1^ and the right P^3^ are missing due to postmortem loss, as is evidenced by the unfilled and sharp-edged alveoli of these teeth. **(d)** Radiograph (lateromedial projection) of the bones of the anterior facial region of specimen ZMUC 139. Note thin walls and wide apical foramen of the right maxillary C as well as marked radiolucency around its root apex (asterisk) indicative of osteolysis in the course of a periapical inflammation.

On macroscopic and radiographic inspection, the five pathological grey seal skulls showed a similar spectrum of dental and bone lesions. All specimens exhibited openings into the pulp cavity at the crown tips of incisors and canines, and in one case (ZMUC 139) also of three anterior premolars (Table 1, Fig 3). Except for one specimen (ZMUC 15^5^, both maxillary C unaffected), exposure of the pulp cavity through an opening at the crown tip was observed in all mandibular and maxillary canines from the five pathological skulls. In the affected teeth, both the tip of the enamel cap and the dentin overlying the pulp horn had thus been lost soon after eruption due to wear. No indication of fracturing of the tooth tips was observed. Except for specimen ZMUC 139, no exposure of the pulp cavity was recorded in postcanine teeth.

**Fig 4:**
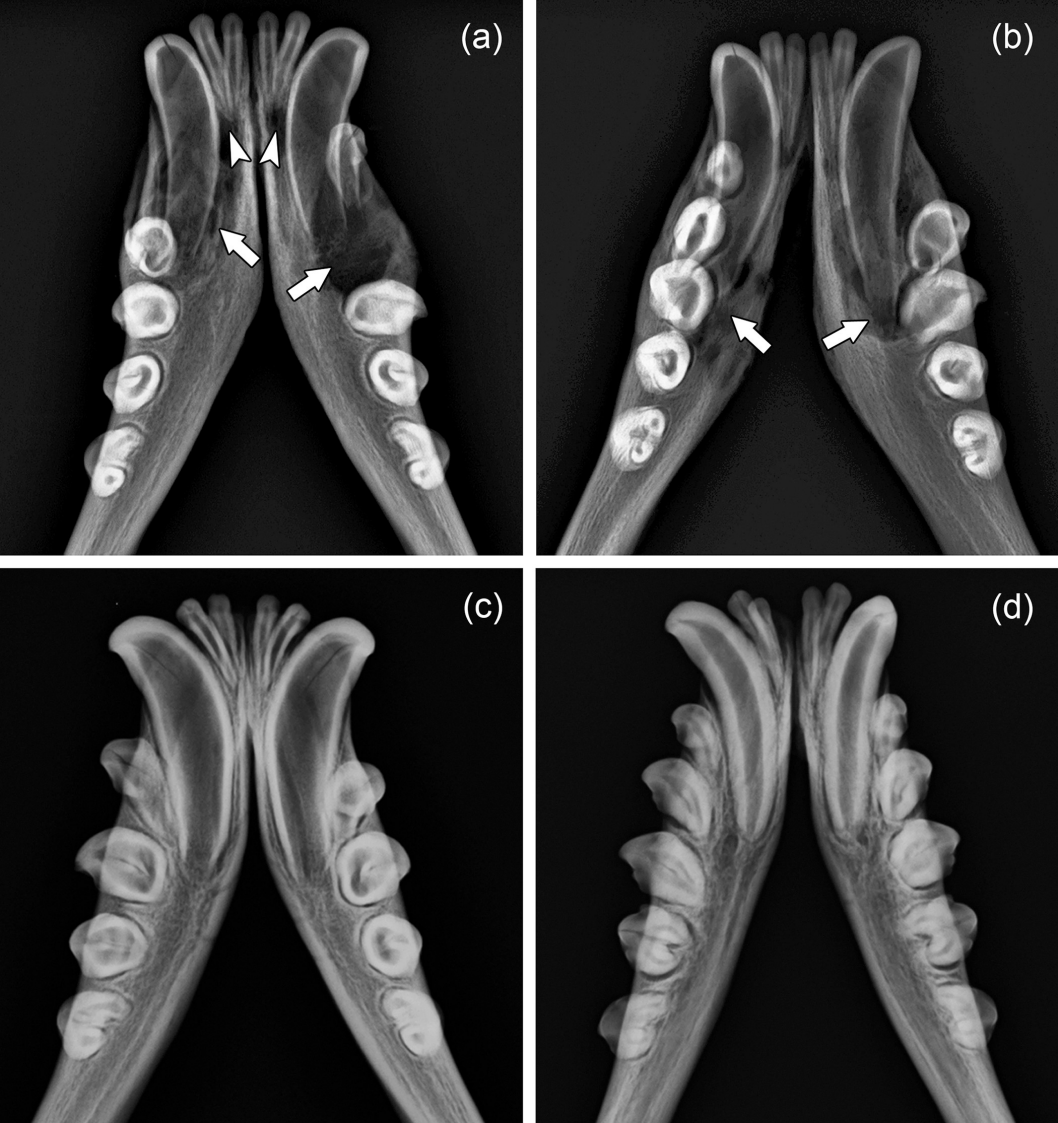
Radiographs (dorsoventral projections) of the anterior portions of the mandibles of four juvenile Baltic grey seals (*Halichoerus grypus grypus*). Note thin walls of the mandibular C exhibiting exposed pulp cavities through openings at the crown tips in the two specimens shown in **(a)** [ZMUC 42^4^] and **(b)** [ZMUC 303] compared to thicker canine walls in the unaffected “control” specimens shown in **(c)** [ZMUC 71^3^] and **(d)** [ZMUC CN 1157]. Arrows: radiolucent areas around the roots of the canines, indicative of pronounced periapical osteolysis. Arrowheads: radiolucency around the root apices of incisors.

In the skulls of the young-of-the-year, radiographic inspection demonstrated that canines with openings at the crown tips had thinner dentin than the unaffected canines of the two controls (Fig 4).

Thickness of the canine walls in the control skull (ZMUC CN 1157; Fig 4D) from the older juvenile was markedly higher (due to progressive dentin apposition) than in the skulls of the young-of-the-year (Fig 4A–4C). In contrast, in pathological specimen ZMUC 139, likewise belonging to an older juvenile, the canine walls were still thin (Fig 3D), denoting a lack of dentin deposition. The canines of the young-of-the-year exhibited wide apical foramina typical for that age (Fig 4A–4C). In the older control specimen (ZMUC CN 1157), the apical foramina of the canines were markedly narrower (Fig 4D), while this was not the case in pathological specimen ZMUC 139 (Fig 3D). The above findings indicate that, following the formation of an initial layer of dentin, no further dentin apposition had occurred in the canines of the pathological skulls. Corresponding changes to those seen in the canines were also observed in the incisors that exhibited openings into the pulp cavity at their crown tips.

**Fig 5:**
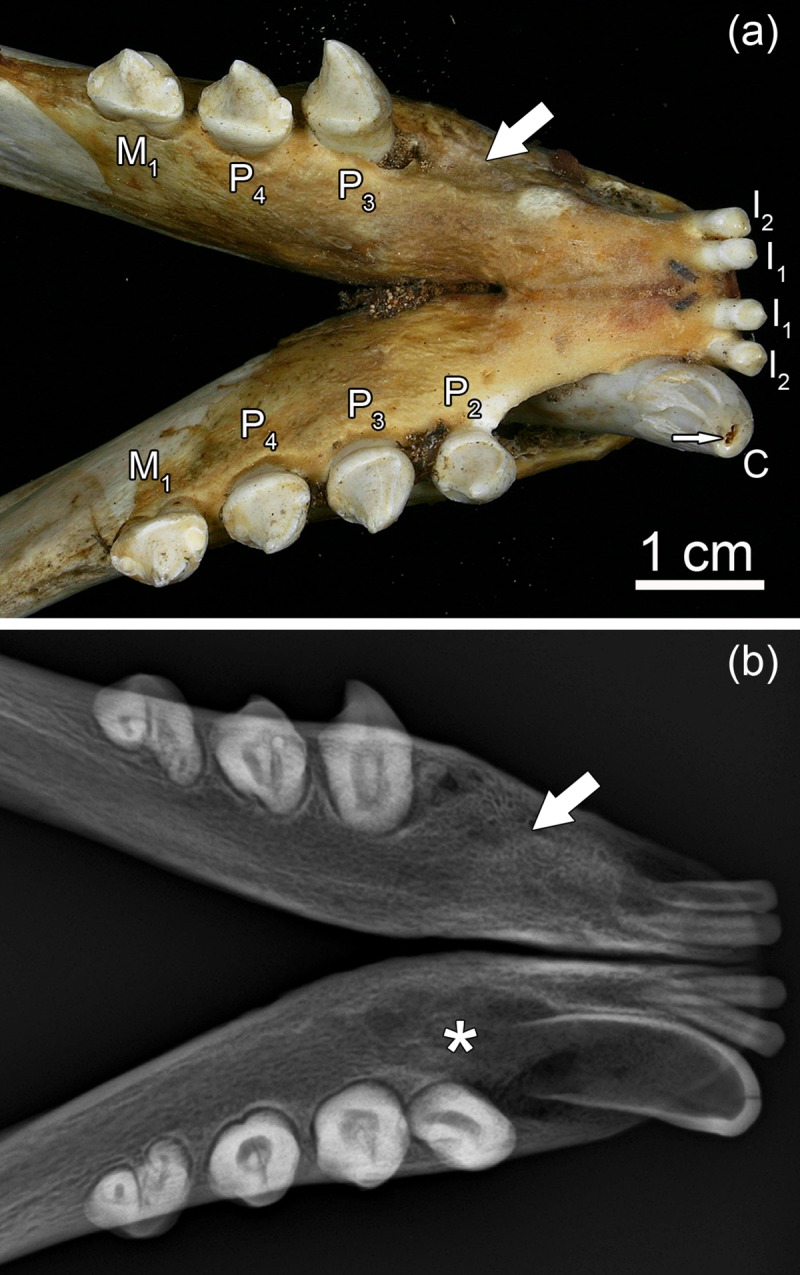
**Dorsal view (a) and radiograph (b) of the anterior mandibular region of a juvenile Baltic grey seal (*Halichoerus grypus grypus*) from the 19th century (specimen ZMUC 15**^**5**^**).** Note opening into the pulp cavity at the crown tip of the right mandibular C (small arrow in **(a)**) and radiolucency indicative of pronounced osteolysis around the root of this tooth (asterisk in **(b)**). In the right mandible, the P_1_ is missing, most likely due to intravital loss. In the left mandible, the C, P_1_ and P_2_ are missing due to intravital loss and the alveoli of these teeth have been filled with bone (large arrows in **(a)** and **(b)**).

In addition to the changes described above, specimen ZMUC 139 showed abnormally intensified wear on all incisors and canines available for inspection as well as on four anterior premolars (right P^1^, both P^2^ and left P_1_). The crowns of the affected teeth had been completely worn away, with only the roots remaining (Fig 3C and 3D). In the pathological skulls (except for specimen ZMUC 139, see above), dentinal thickness of premolars and molars was markedly higher than that of the incisors and canines with exposed pulp cavities (Fig 4A and 4B; Fig 5B).

Radiographic inspection demonstrated pronounced radiolucency around the root apices of the teeth with exposed pulp cavities (Figs 3D, 4A and 4B, 5B), indicating intense bone resorption in the course of an inflammatory process. In the left mandible of specimen ZMUC 15^5^, the canine and the P_1_ and P_2_ were missing (intravital loss), and the alveoli of these teeth had become partially (C) or completely (P_1_ and P_2_) filled with new bone (re-ossification of alveoli) (Fig 5).

**Fig 6:**
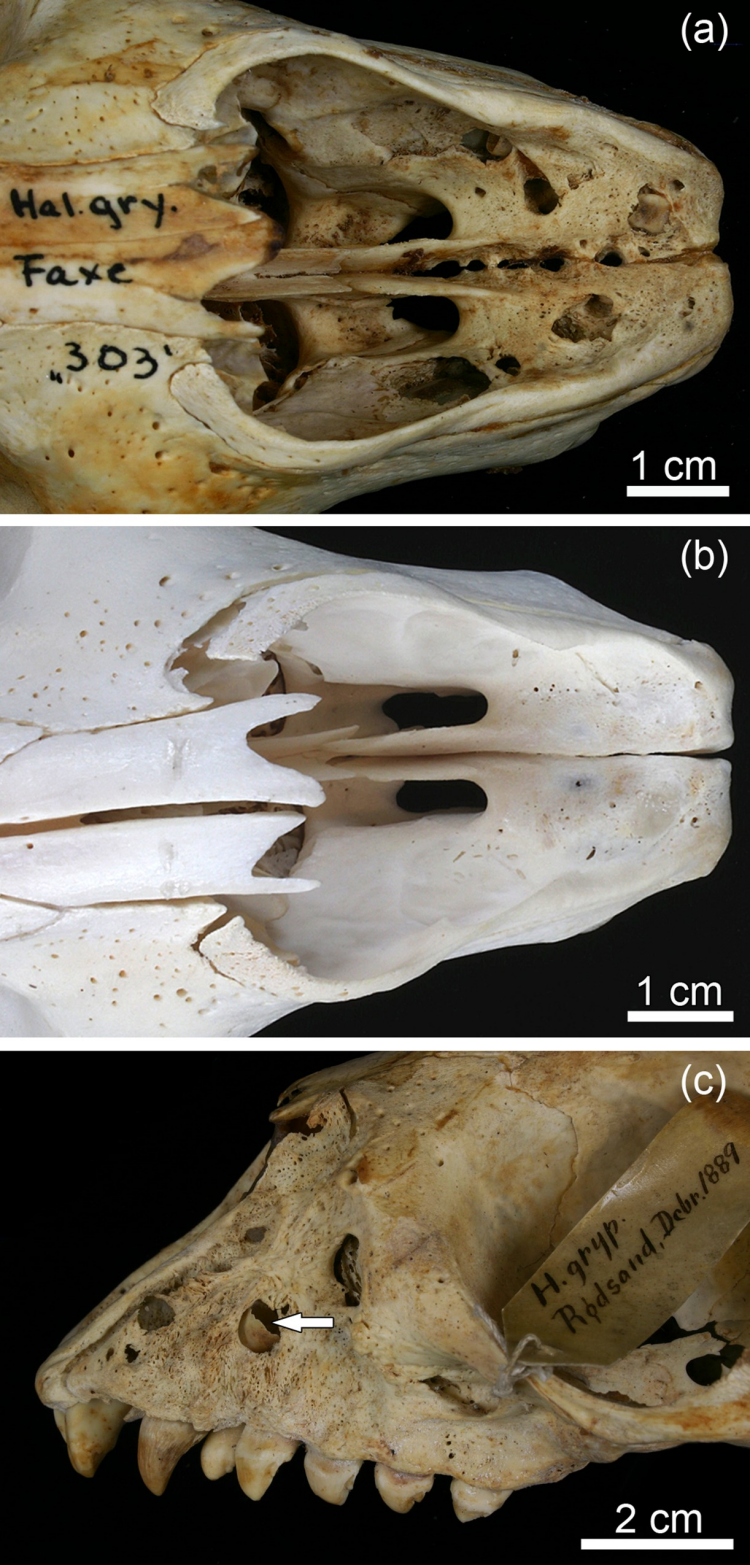
Osteolytic lesions in the skulls of juvenile Baltic grey seals (*Halichoerus grypus grypus*). **(a)** Dorsal view of the anterior cranium of a juvenile grey seal from the 19th century (specimen ZMUC 303) showing numerous openings of draining tracts in the incisive bones. **(b)** Dorsal view of the anterior cranium of a modern juvenile grey seal (specimen ZMUC CN 1157) free of such lesions. **(c)** Left lateral view of the anterior cranium of a juvenile grey seal from the 19th century (specimen ZMUC 69^3^) showing openings of draining tracts and eroded surface of the maxilla indicative of extended cortical bone resorption. Arrow: root tip of left maxillary C.

**Fig 7:**
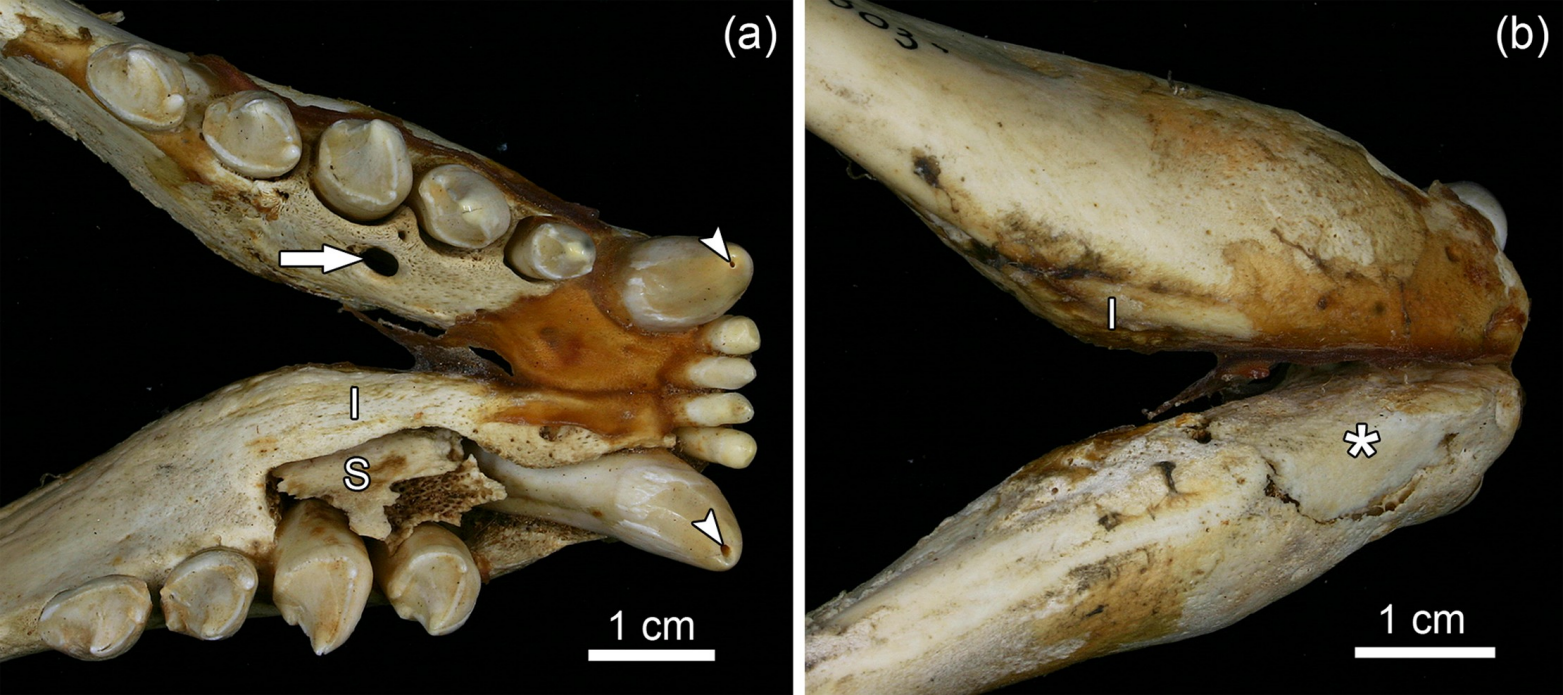
**Dorsal (a) and ventral (b) views of the anterior portion of the mandibles of a juvenile Baltic grey seal (*Halichoerus grypus grypus*) from the 19th century (specimen ZMUC 303) showing signs of suppurative osteomyelitis. (a)** Note opening of a draining tract in the left mandible (arrow), and presence of a large (entirely detached) sequestrum (S) and an involucrum (I) in the right mandible. Arrowheads: openings into the pulp cavities at the tips of both mandibular C. The right P_1_ is missing, most likely due to intravital loss. **(b)** Note presence of a large sequestrum (asterisk), demarcated by a dividing line but still in place, in the left mandible, and occurrence of an involucrum (I) in the right mandible. An incomplete pathological fracture of the left mandible had occurred along the demarcation line.

**Fig 8:**
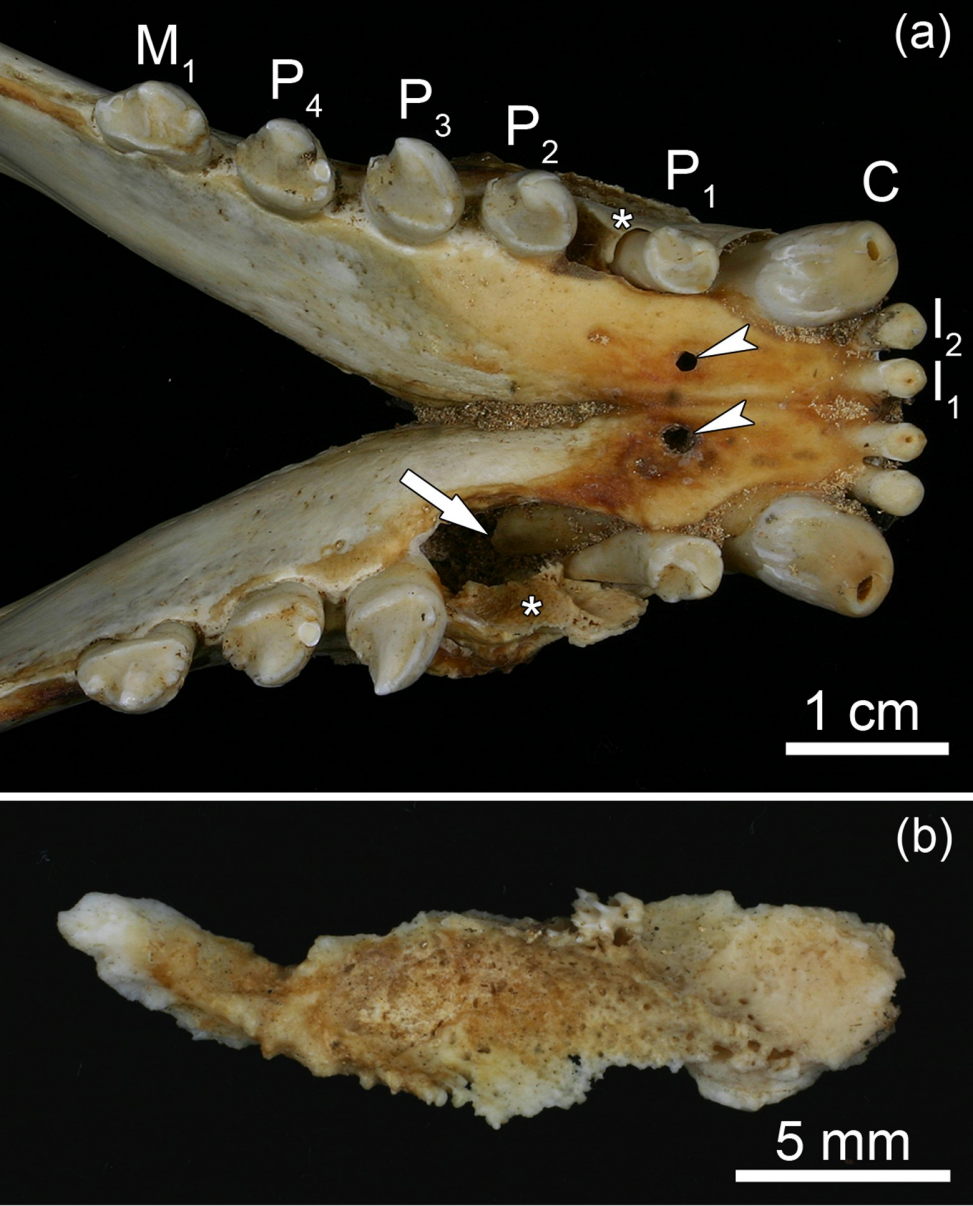
Signs of periapical abscesses and suppurative osteomyelitis in the mandibles of a juvenile Baltic grey seal (*Halichoerus grypus grypus*) from the 19th century (specimen ZMUC 42^4^). **(a)** Dorsal view of anterior mandibular region. Note openings into the pulp cavities at the crown tips of both I_1_ and both mandibular C, openings of draining tracts (arrowheads), large resorption cavity around the root of the right canine and presence of a large sequestrum in each mandible (asterisks). The loose right P_2_ was removed to show the resorption cavity. **(b)** Completely detached sequestrum that was removed from the right mandible. The surface of the sequestrum shows traces of intense resorption.

Occurrence of the extended osteolytic lesions around the root apices was associated with a spectrum of other pathological changes in the incisive, maxillary, and mandibular bones (Figs 6 – 8). These changes included areas of extensive cortical bone destruction and openings of draining tracts for pus discharge in the cortex (Fig 6). In addition, the bilateral presence of sequestra and involucra was observed in the mandibles of two of the pathological specimens (ZMUC 42^4^ and ZMUC 303) (Figs 7 and 8). In specimen ZMUC 303, an incomplete pathological fracture had occurred in the left mandibular corpus along the demarcation line around the sequestrum (Fig 7B).

**Fig 9:**
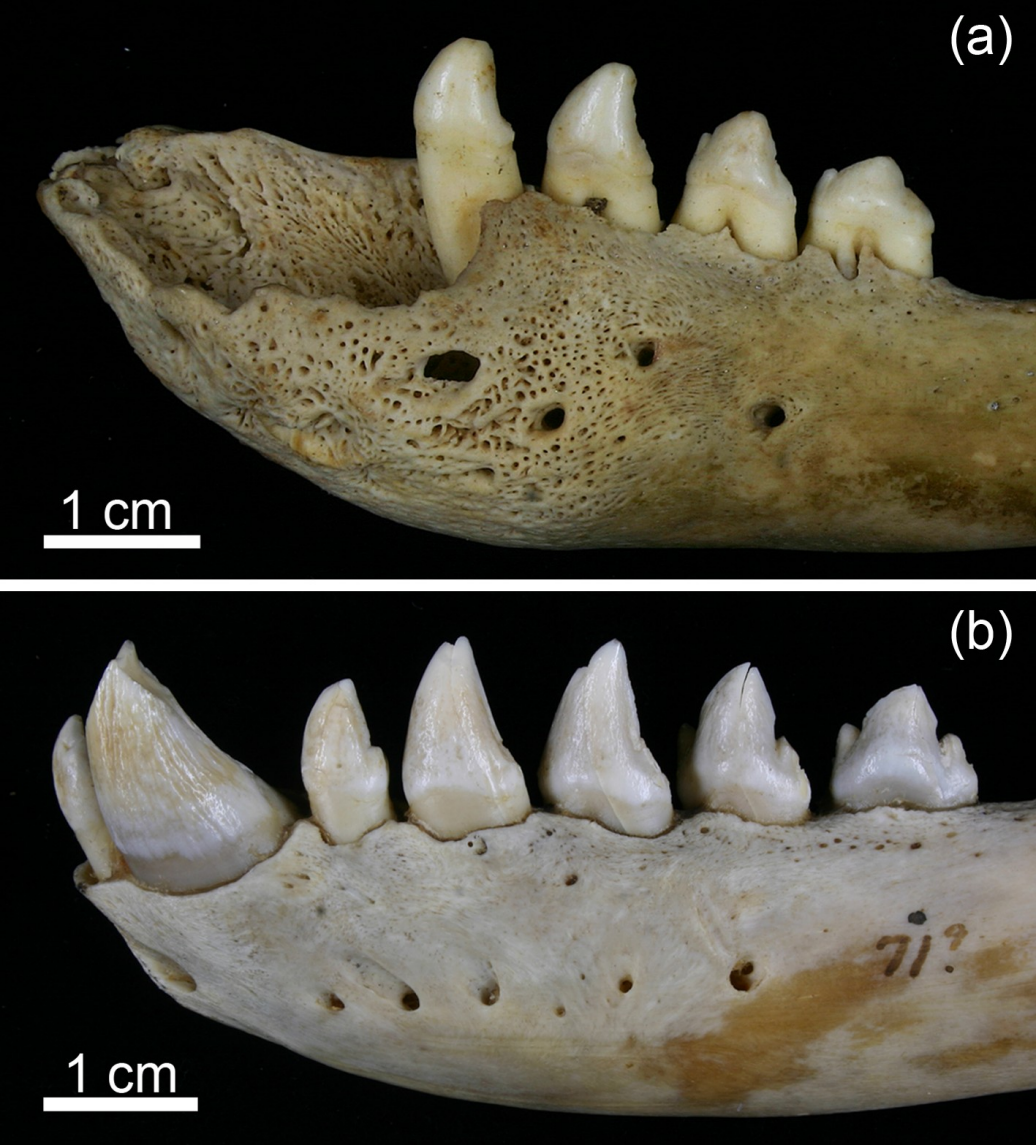
Lateral views of the anterior regions of the left mandibles of two juvenile Baltic grey seals (*Halichoerus grypus grypus*) from the 19th century. **(a)** Specimen ZMUC 139; I_1_, I_2_, C and P_1_ are missing, most likely due to intravital loss. Note widened alveolus of the mandibular C and enlargement of the mandible due to apposition of periosteal new bone with numerous small vascular foramina. Porous bone has also been deposited on the wall of the enlarged alveolus. Some of the mental foramina appear enlarged. The changes are indicative of a severe inflammatory process in the anterior mandible. **(b)** Specimen ZMUC 71^9^, showing the normal condition of the mandible for comparison.

The anterior portions of the mandibles of the five pathological specimens were enlarged due to the apposition of porous periosteal new bone (Fig 9). This is evidence of a proliferative reaction of the periosteum to the inflammatory process. Porous bone apposition was also noted on the walls in several of the widened alveoli of incisors and canines (Fig 9A).

**Fig 10:**
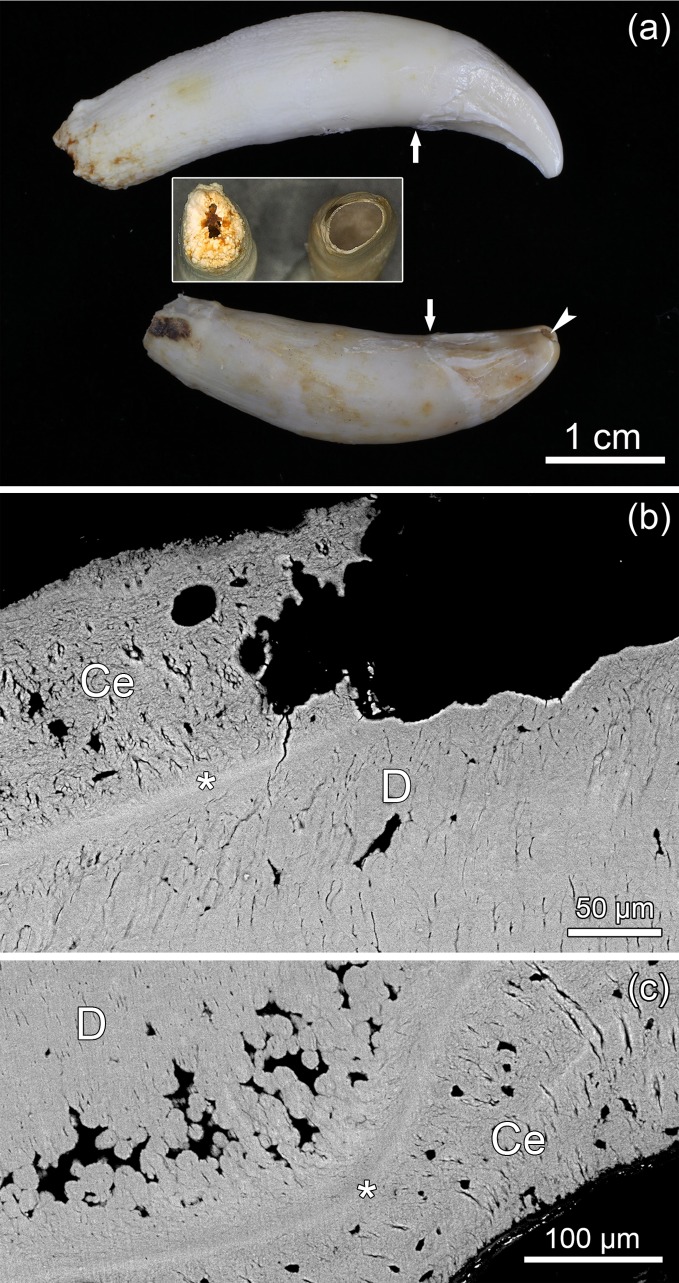
Permanent maxillary canines of juvenile Baltic grey seals (*Halichoerus grypus grypus*). **(a)** Left maxillary C of specimen ZMUC CN 1157 (top) and right maxillary C of specimen ZMUC 69^3^ (bottom). Root development is more advanced in the canine of ZMUC CN 1157. Arrows: crown-root border; arrowhead: opening into the pulp cavity at the crown tip of the right maxillary C of ZMUC 69^3^. Insert: apical foramina of the canines of ZMUC CN 1157 (left) and ZMUC 69^3^ (right). **(b)** SEM-BSE image of polished cut surface of the longitudinally sectioned left maxillary C of specimen ZMUC 69^3^, showing external root resorption affecting cementum (Ce) and dentin (D). Asterisk: cementum-dentin-junction. **(c)** SEM-BSE image showing interglobular dentin (areas where the calcospherites have failed to fuse; these areas appear black in the image) in the root of the left maxillary C of specimen ZMUC 69^3^. Ce: cementum; D: dentin; asterisk: cementum-dentin-junction.

**Fig 11:**
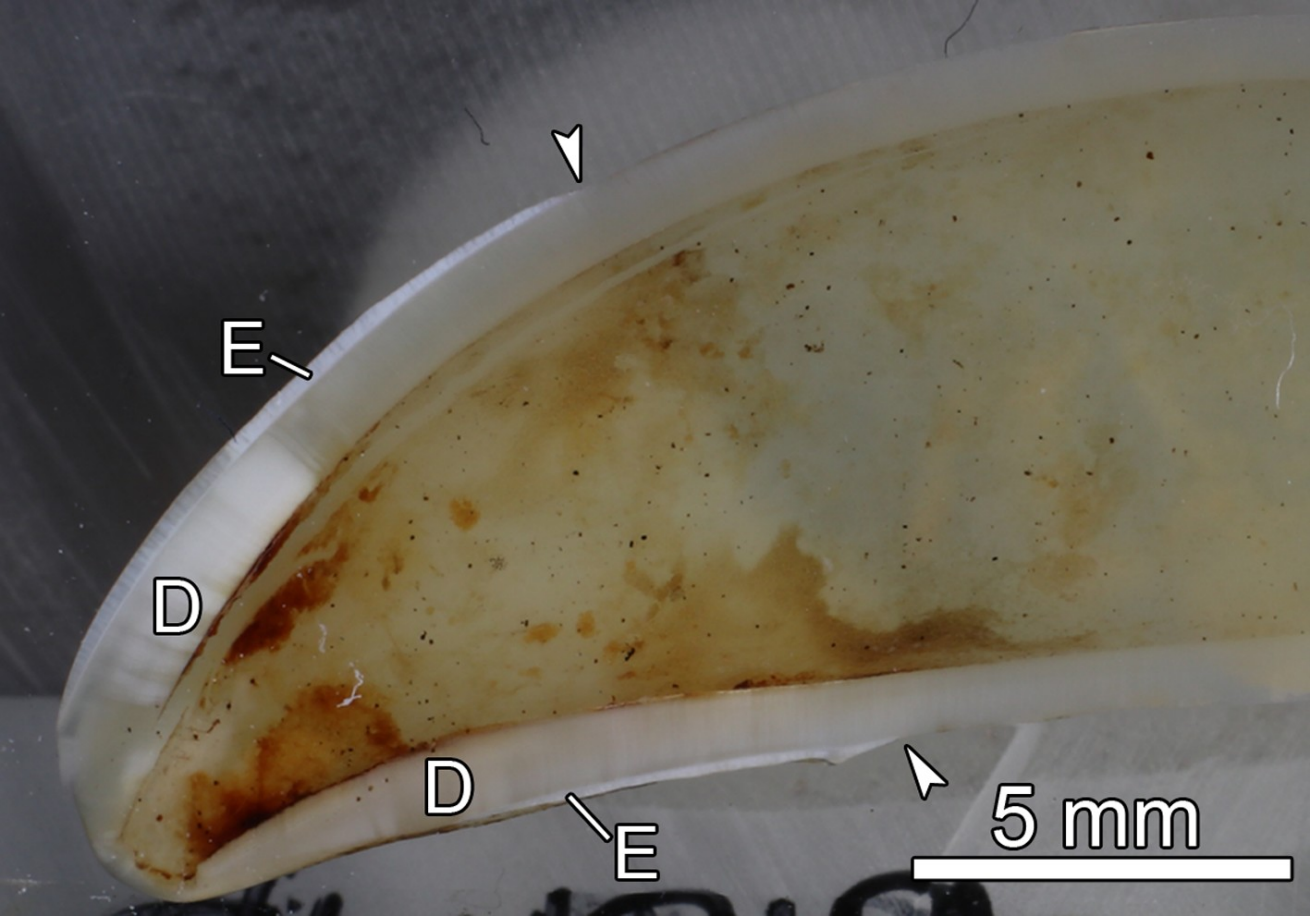
Longitudinally sectioned right maxillary C of specimen ZMUC 69^3^. Exposure of the pulp cavity through an opening at the crown tip. D: dentin; E: enamel; arrowheads: crown-root border. Resin-embedded tooth; labial side directed to upper-left corner of image.

The two extracted maxillary canines showed a similar position of the crown-root border (Fig 10A). While the enamel cover of the maxillary canine of the control specimen ZMUC CN 1157 was still present, it had been worn away in the incisal area of the maxillary canine of specimen ZMUC 69^3^ (Fig 10A, Fig 11). Following the wearing away also of the incisal primary dentin, pulp exposure had occurred in the former tooth.

**Fig 12:**
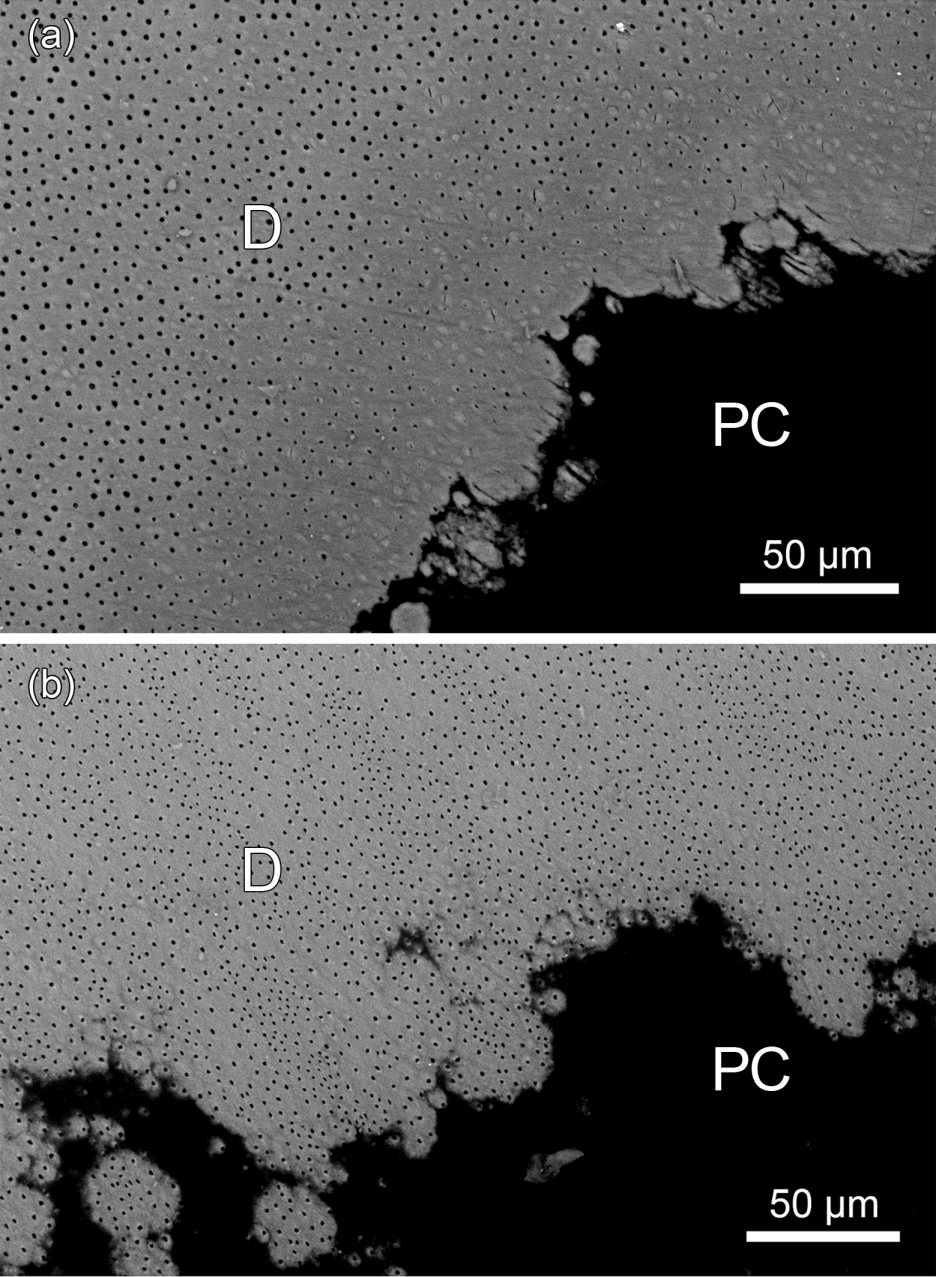
SEM-BSE images of polished cut surface showing the dentin overlying the pulp cavity in permanent maxillary canines of juvenile Baltic grey seals (*Halichoerus grypus grypus*). **(a)** Right maxillary C of specimen ZMUC 69^3^. Note occlusion of many dentinal tubules in juxtapulpal dentin by mineral deposits. As evidenced by its higher brightness, the material occluding the dentinal tubules is more highly mineralized than the surrounding intertubular dentin. PC: resin-filled pulp cavity. **(b)** Left maxillary C of specimen ZMUC CN 1157. No occlusion of dentinal tubules. D: dentin; PC: resin-filled pulp cavity.

**Fig 13:**
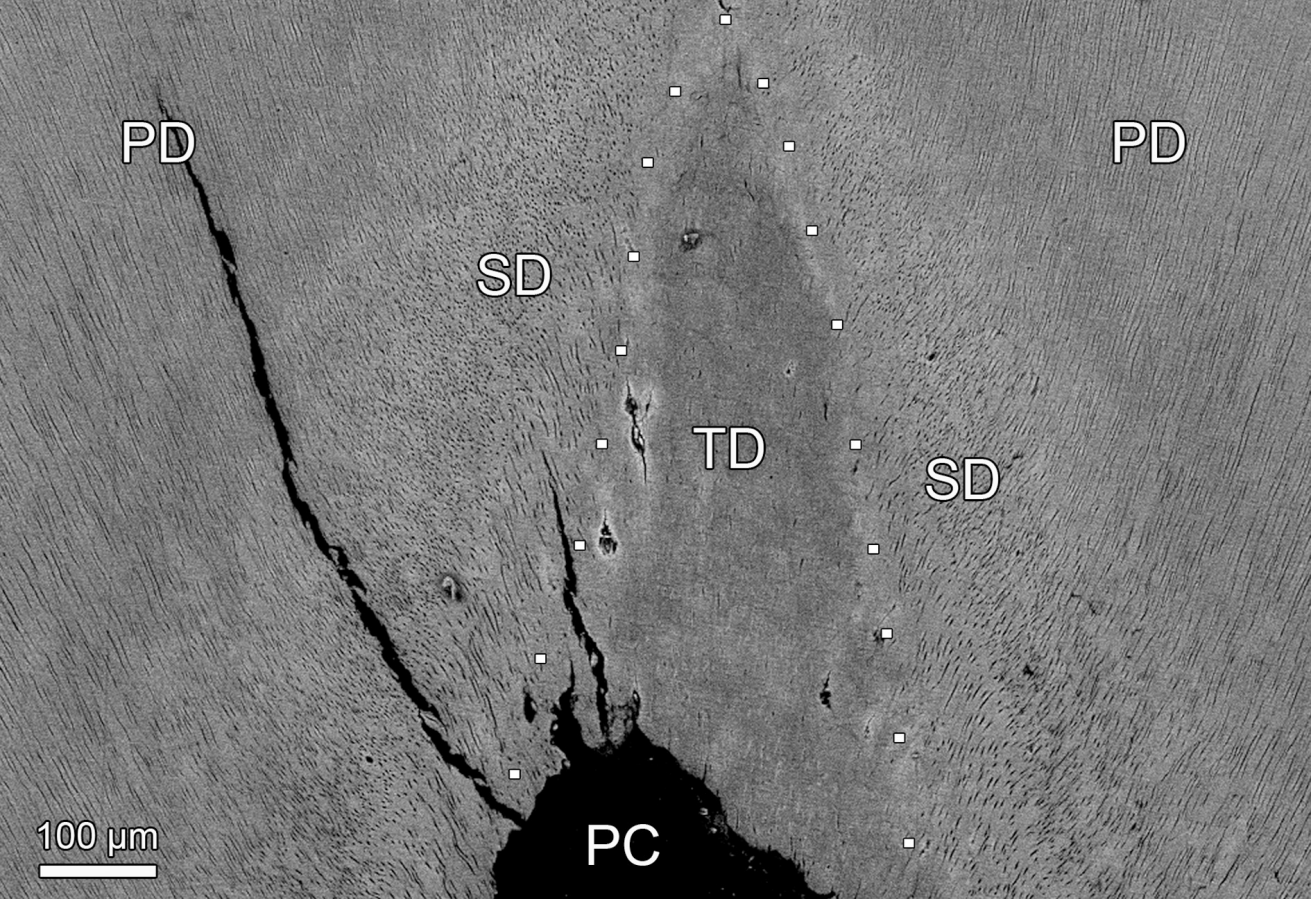
SEM-BSE image of polished cut surface of the bucco-lingually sectioned M_1_ of specimen ZMUC 69^3^ showing the dentin overlying the pulp cavity. Three types of dentin can be distinguished. The primary dentin (PD) is characterized by regularly arranged, straight dentinal tubules, whereas the tubules in the secondary dentin (SD) are curved and arranged somewhat more irregularly. The tertiary dentin (TD) that has been deposited in the pulp horn exhibits only very few tubules. PC: resin-filled pulp cavity. The stippled line indicates the approximate border between secondary and tertiary dentin.

SEM-BSE imaging revealed that in the juxtapulpal dentin overlying the pulp cavity in the canine of specimen ZMUC 69^3^ many dentinal tubules had become occluded by mineral deposits (Fig 12A), while this was not the case in the canine of control specimen ZMUC CN 1157 (Fig 12B). The overall density and arrangement of the dentinal tubules in coronal dentin did, however, not differ between the two teeth. SEM-BSE imaging further demonstrated signs of external and internal root resorption in the canine of specimen ZMUC 69^3^. The marked external root resorption had affected both cementum and dentin (Fig 10B). Signs of internal resorption were present in the dentin of the root canal near the root apex. Contrary to the control canine, the root dentin of the canine of ZMUC 69^3^ also exhibited rather extended areas of interglobular dentin (Fig 10C). In contrast to the canine, the analyzed M_1_ of ZMUC 69^3^ exhibited both secondary and tertiary dentin in its crown (Fig 13) that still possessed a complete enamel covering. The tertiary dentin had been deposited in the tip of the pulp cavity and was largely atubular. Corresponding findings were made in the studied M_1_ of specimen ZMUC 71^9^.

**Fig 14:**
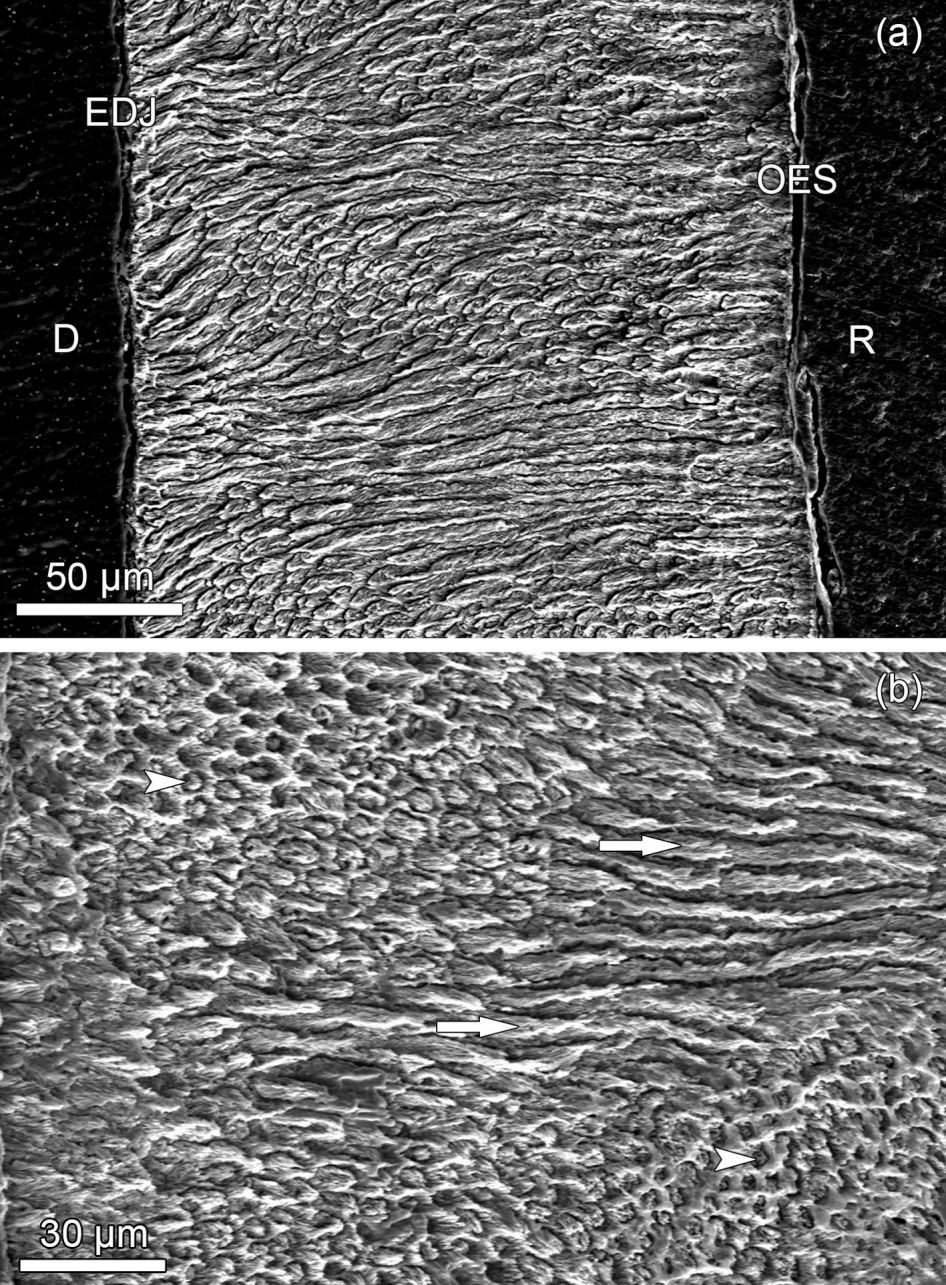
Enamel structure in longitudinally sectioned teeth of specimen ZMUC 69^3^; SEM-SE images of etched polished cut surface. **(a)** buccal enamel of the right M_1_. The enamel is prismatic and shows Hunter-Schreger bands, i.e., alternating zones of more longitudinally and more transversely sectioned prisms. D: dentin; EDJ: enamel-dentin junction, OES: outer enamel surface; R: resin. **(b)** labial enamel of the right maxillary C showing zones with transversely (arrowheads) and longitudinally (arrows) sectioned prisms. The outer enamel surface is located to the left of the field of view.

SEM-SE imaging of etched cut surfaces revealed normal prismatic structure and presence of Hunter-Schreger bands in the enamel of all four analyzed teeth (Fig 14). Hardness values of enamel and coronal dentin did not differ significantly between the two studied canines (Table 2).

**Table 2:** Vickers hardness of enamel and coronal dentin in maxillary canines of two juvenile Baltic grey seals, *Halichoerus grypus grypus* (left maxillary C of specimen ZMUC CN 1157, right maxillary C of specimen ZMUC 69^3^).

Specimen	Hardness number (HV 0.025)	
	Enamel [median; mean (SD)]	Dentin [median; mean (SD)]
ZMUC CN 1157	166.5; 174.1 (29.6)	48.8; 49.0 (6.5)
ZMUC 69^3^	171.0; 176.8 (28.4)	54.2; 55.9 (8.0)
P-value	0.79 (ns)	0.11 (ns)

P-values are from Mann-Whitney U-tests; Eight measurements each in enamel and dentin per tooth; SD = standard deviation; ns = not significant.

## Discussion

In the five grey seals exhibiting pathological skull changes, pulp exposure of incisors, canines and, in one skull also anterior premolars had occurred following the loss of the enamel layer covering the crown tip and the wearing away of the subjacent thin dentin layer overlying the pulp cavity. Normally, pulp exposure due to tooth wear is prohibited by the lifelong apposition of dentin that causes a progressive narrowing of the pulp cavity with age [22].

The fact that, except for specimen ZMUC 139, only incisors and canines showed pulp exposure might be linked to the feeding mode of the grey seal. During the piercing bite used for prey capturing, the anterior teeth are likely exposed to more intense wear than the posterior ones. It is presently unclear, why in some anterior teeth (e.g., the canine of skull ZMUC 69^3^) the enamel covering the crown tip was lost early, while in others (e.g., the canine of skull ZMUC CN 1157) it was still present. As was demonstrated by SEM imaging, the enamel of both teeth showed a normal microstructure, which corresponded to that reported for other pinniped species [43]. Hardness testing on the two grey seal canines revealed no significant differences in the degree of mineralization of their enamel and dentin. Thus, the quality of their enamel did apparently not differ between the two teeth. Possible factors responsible for an early local loss of enamel in anterior teeth include individual or sex-related variation in enamel thickness and variation in the composition (and related “abrasiveness”) of the animals’ diet.

According to Hewer [22], at birth the canines of grey seals possess a thin cone of prenatally formed primary dentin, which this author refers to as “fetal dentin”. In the first three to four postnatal weeks, while the canines considerably increase in length, a second dentin cone, referred to as “pup dentin”, is laid down onto the prenatally deposited dentin, and subsequently further dentin layers are added in an incremental fashion [22]. Given the early eruption of the permanent teeth in the grey seal, at least the later-formed pup dentin has to be classified as secondary dentin.

In the five pathological specimens, no or only little dentin had apparently been laid down in anterior teeth following the deposition of an initial (primary) dentin layer. This failure to deposit (sufficient) secondary and/or tertiary dentin in response to wear led to exposure, infection, inflammation (pulpitis) and, finally, necrosis of the pulp in these teeth. In contrast, the postcanine teeth of the pathological skulls typically had thick dentinal walls and showed no pulp exposure. Furthermore, contrary to the studied canine from this skull, the analyzed M_1_ of specimen ZMUC 69^3^ exhibited deposition of secondary and tertiary dentin at the tip of the pulp cavity, even though the coronal dentin of this tooth was still covered by enamel. The thinner dentin in the canines of the three pathological skulls from young-of-the-year compared to those of the two control skulls from this age group indicates a rapid death of the pulp following its early exposure in the former that then precluded any further dentin formation.

The higher dentinal thickness in postcanine teeth of the pathological skulls suggests that the reason for the observed pathological condition may have been a late onset of secondary and tertiary dentin formation rather than the absolute inability to produce these dentin types. According to this view, for posterior teeth this was compatible with maintaining a viable pulp and ongoing dentin apposition, whereas it proved fatal for anterior teeth in which more intense wear led to pulp exposure and necrosis.

In human dentistry, occlusion of dentinal tubules by mineral deposits, as was observed in the juxtapulpal coronal dentin of the maxillary canine of specimen ZMUC 69^3^, is a typical response of the pulpodentinal complex to caries lesions, particularly slowly progressing and arrested ones [44]. Tubular occlusion causes a reduction in dentin permeability, thereby slowing down further lesions progression [44]. Another potential stimulus that can cause occlusion of dentinal tubules is increased dental attrition [45, 46]. Rapid wear of the grey seal teeth with only thin dentinal walls was substantiated in the present study, and it is suggested that the occlusion of dentinal tubules occurred as a response to this stimulus.

In teeth of juvenile grey seals (≤ 1 year), formation of interglobular dentin has not been reported [22]. In the harp seals (*Phoca groenlandica*), layers of interglobular dentin are frequently part of later-formed annual growth layer groups, but not in that representing the first year of growth [47]. The interglobular dentin present in the canine of specimen ZMUC 69^3^, which was formed in the year of birth, is therefore considered a pathological feature.

In the five pathological grey seals, the inflammation had spread from the pulp into the periapical space, causing severe lytic lesions with extensive destruction of periapical bone, radiographically visible as extended radiolucent spaces around the tooth roots, and the formation of draining tracts for pus discharge. Especially via the large, deeply inserted canines with their still very wide apical foramina (typical for juvenile seals) the inflammation could easily spread into the jaws and cause the formation of periapical abscesses. The findings in the maxillary canine from specimen ZMUC 69^3^ indicate pronounced external resorption on the tooth root situated in the abscess cavity, the resorptive process penetrating from the cementum into the dentin. The canine also showed signs of internal root resorption in the course of the inflammatory process, which is a common finding in teeth with periapical lesions [48].

From the periapical space the inflammation extended into the surrounding bone regions, leading to suppurative osteomyelitis of the jaws. The bone lesions formed in the course of the osteomyelitis include perforations and extensive subperiosteal resorption of cortical bone, periosteal new bone formation due to proliferative periostitis, and the formation of sequestra and involucra in the mandibles of two animals. Formation of sequestra, i.e., of pieces of bone that have become necrotic due to ischemia and are separated from the surrounding living bone, is a typical sign of osteomyelitis [49–52]. An involucrum is a layer of living bone formed around a sequestrum by periosteal activity [49, 50]. The young age of the pathological specimens indicates a rapid progression of the disease process once pulp exposure of the teeth had occurred. It is concluded that the affected individuals had suffered from severe septicemia, which would probably have resulted in their early death.

Periapical abscesses associated with (mostly maxillary) canines and maxillary second incisors have been reported in Weddell seals (*Leptonyches weddellii*) following exposure of the pulp cavity by excessive wear [53]. This excessive wear was related to the use of the teeth for abrading sea ice to keep breathing holes open during winter. In contrast to the situation in the grey seals studied by us, exposure of the pulp cavities of canines and incisors in the Weddell seals occurred in older animals in which dentin formation had progressed normally [53]. Periapical abscesses and osteomyelitis of the jaws secondary to exposure of the pulp cavities can also occur if teeth are rapidly worn down due to a hypomineralization of their enamel, as has for instance been observed in wild red deer (*Cervus elaphus*) exhibiting dental fluorosis [54]. In the grey seals, hardness testing indicated no significant difference in the degree of mineralization of enamel and coronal dentin between the pathological and control canine.

Defective dentinogenesis characterizes different dental disorders, and it may therefore be asked, whether the condition seen in the pathological grey seal teeth can be related to specific disorders known in dental pathology. Odontodysplasia is a rare, non-hereditary developmental disturbance of uncertain etiology in humans [55–58]. As the anomaly is usually of a localized nature, affecting mostly teeth of only one quadrant of the dentition, it is mostly referred to as regional odontodysplasia. A corresponding condition has also been described in a maxillary canine of a domestic dog [59]. Affected teeth show wide pulp cavities and thin dentin with occurrence of interglobular dentin [55–58]. However, in odontodysplasia also the enamel is abnormal, exhibiting both hypoplasia and hypomineralization [55–58]. In contrast, the enamel of the affected canine of specimen ZMUC 69^3^ was normally mineralized, and none of the teeth of the pathological grey seal skulls exhibited enamel hypoplasia. The pathological changes in the grey seal teeth are therefore not consistent with a diagnosis of regional odontodysplasia.

In humans, teeth with enlarged pulp cavities and only thin dentin layers, referred to as shell teeth, have been reported from individuals with dentinogenesis imperfecta type III (DGI-III), a heritable dentin defect with an autosomal dominant mode of inheritance [60–64]. The condition is associated with a high frequency of pulp exposure and occurrence of periapical radiolucencies. SEM observations on permanent teeth of patients with DGI-III revealed normal enamel structure, while the dentin showed structural changes [61]. DGI-III is associated with mutations in the *DSPP* gene that encodes dentin sialophosphoprotein (DSPP), a non-collagenous extracellular matrix protein [62–65]. The DSPP produced by the odontoblasts is proteolytically cleaved into fragments, thereby converting the inactive precursor into active proteins involved in the formation and mineralization of dentin [63–67].

*DSPP*^-/-^ knockout mice (*Mus musculus*) exhibit dental defects similar to those seen in humans with DGI-III. The affected mouse teeth have enlarged pulp cavities, pulp exposure and degeneration, periapical bone resorption and abnormally intensified wear [65]. Light and scanning electron microscopy revealed a marked reduction in the thickness of mineralized dentin, a widened predentin zone, an irregular dentinal mineralization front, and the frequent occurrence of interglobular dentin in the teeth of the *DSPP*^-/-^ mice [65]. Similar changes and a marked drop in dentinal mineral apposition rate compared to controls were also observed in mice in which the proteolytic processing of DSPP had been blocked [67].

Contrary to the presentation of DGI-III in humans and the dental changes seen in the DSPP^-/-^ knockout mice, in the pathological grey seals only anterior teeth showed thin dentin and enlarged pulp cavities. This is in our view not consistent with the diagnosis of a pinniped equivalent of DGI-III. However, it could be speculated that the condition in the grey seals was related to a less severe disturbance in the formation, secretion, or processing of DSPP that caused a delay in dentin apposition following the formation of an initial primary dentin layer.

The reported dental and skull pathology has thus far been observed only in juvenile grey seals collected in 1889/1890 and within a relatively narrow area of the subspecies, which might be seen as circumstantial evidence for a heritable dentin defect in the animals. Future studies are needed to clarify a possible genetic causation of the reported condition in the Baltic grey seals. The fact that the condition is not known from modern grey seals could mean that it does not exist in today’s populations, although the possibility exists that it is only unreported. Therefore, further studies addressing dental pathology in other grey seal populations are encouraged.

It has been experimentally demonstrated in rats (*Rattus norvegicus*) that certain persistent organic pollutants can disturb tooth development and impair dentinogenesis [68–72]. However, in the late 19th century these substances were not yet existing and the Baltic Sea was still unpolluted [73]. Therefore, pollutant exposure is excluded as a factor involved in the formation of the lesions observed in the grey seals from that period.

In conclusion, the present study has demonstrated the occurrence of a specific and previously unreported form of dental defect of unknown etiology and its sequelae (pulp exposure and inflammation, periapical abscesses and osteomyelitis of the jaws) in Baltic grey seals from the 19th century. It is hypothesized that a late onset or reduced rate of secondary and tertiary dentin formation led to pulp exposure in anterior teeth that were exposed to more intense wear than posterior ones. The present study underscores the importance of systematic studies of museum collections for a broadening of our knowledge of pathological dental and skeletal conditions in wild mammals.

## Supporting information

S1 Fig Right lateral view of the skull of a juvenile male Baltic grey seal (Halichoerus grypus grypus; specimen ZMUC CN 1157). (TIF)
