# Supplementary figures and images for: Dental pulp exposure, periapical inflammation and suppurative osteomyelitis of the jaws in juvenile Baltic grey seals (Halichoerus grypus grypus) from the late 19th century
Source: PLoS One. 2019 Apr 12;14(4):e0215401. doi: 10.1371/journal.pone.0215401 (PMC6461278; doi:10.1371/journal.pone.0215401)

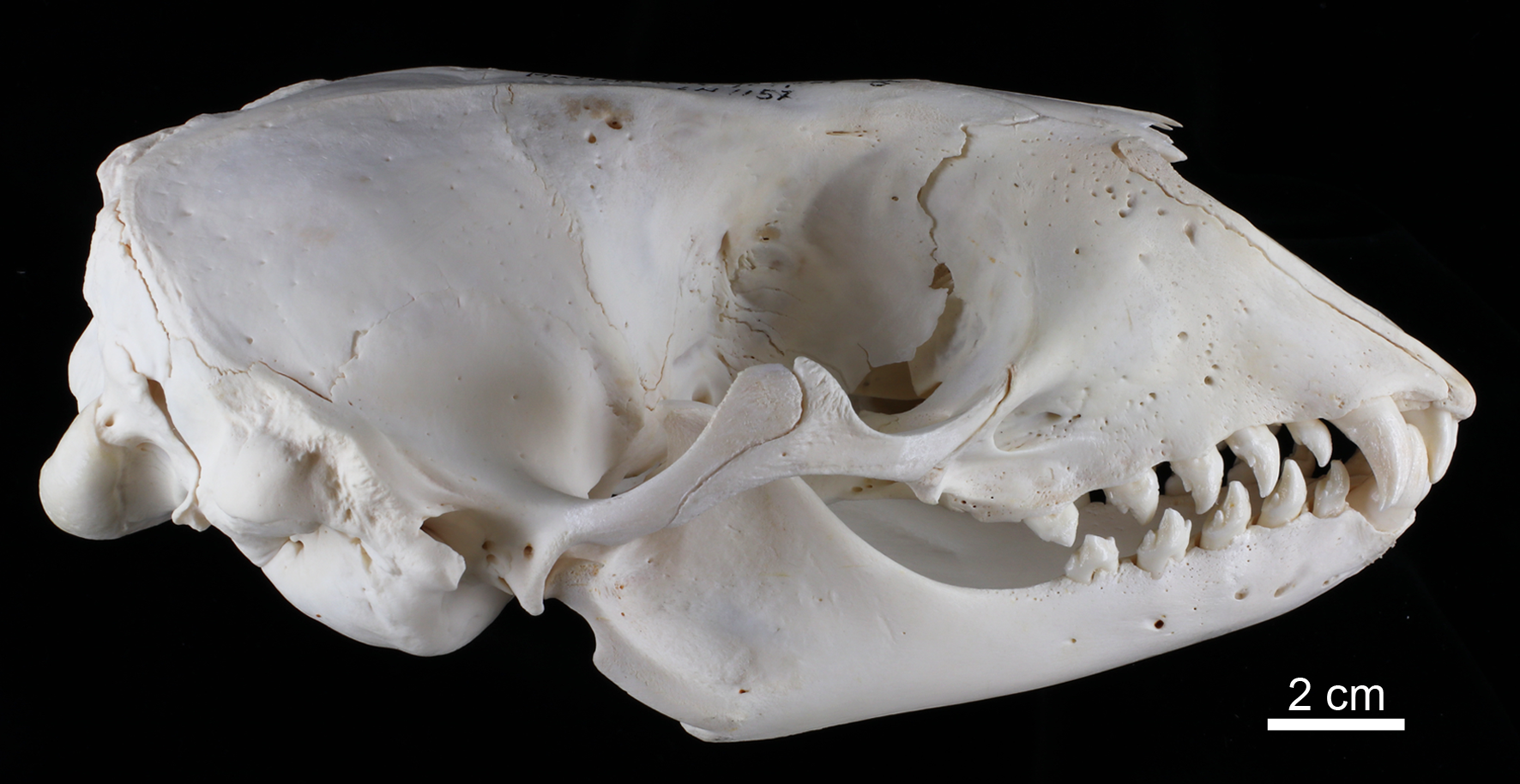

Supplement: S1 Fig — (TIF) [file pone.0215401.s001.tif]
